# Immunogenicity of Hantavirus and evaluation of vaccine protective efficacy: A comparative neutralizing antibody study of vaccine strains and representative epidemic strains from selected provinces in China

**DOI:** 10.1371/journal.pntd.0014811

**Published:** 2026-11-11

**Authors:** Xiaoxia Huang, Zhangqi Zheng, Shanshan Du, Aqian Li, Fangzhen Tian, Tianmeng Gu, Lanling Zheng, Li Chuan, Qin Wang, Li Yuan, Jiandong Li

**Affiliations:** 1 National Key Laboratory of Intelligent Tracking and Forecasting for Infectious Diseases, Beijing, People’s Republic of China; 2 National Institute for Viral Disease Control and Prevention, Chinese Center for Disease Control and Prevention, Beijing, People’s Republic of China; 3 School of Public Health, Key Laboratory of Environmental Pollution Monitoring and Disease Control, Ministry of Education, Guizhou Medical University, Guiyang, Guizhou, People’s Republic of China; Air Force Medical University, CHINA

## Abstract

**Background:**

Hemorrhagic fever with renal syndrome (HFRS) caused by Hantavirus (HV) remains a significant public health burden in the world. HFRS bivalent inactivated vaccine (HFRSbiv) with Hantaan virus (HTNV) and Seoul virus (SEOV) strains isolated 20 years ago have been used in key HFRS endemic provinces in China for HFRS prevention. After decades of effective implementation of comprehensive prevention and control measures, HFRS has entered a stage of epidemic fluctuation at low-level in China. However, the continuous transmission of the virus among rodent host animals may lead to dynamic changes in genetic diversity and immunogenicity of the virus, which may affect the pathogenic mechanism of the virus and the protective effect of vaccines. It is necessary to carry out timely monitoring and evaluation of the immunogenicity of HV.

**Methodology/principal findings:**

A panel of sera was established by screening serum samples from HFRS patients at convalescent phase and individuals who had received standardized immunization with HFRSbiv. Neutralizing antibody titers against HTNV vaccine strain 84FLi, SEOV vaccine strain L99, and three representative epidemic strains from different genotypes (HTNV: SX26, SEOV: LN03 and FJ-17) were measured using an enzyme-linked immunosorbent assay-based microneutralization test (MN-ELISA). HV-neutralizing antibodies were detected from all the selected serum samples. Among HFRS patients, 47.92% (23/48) were identified as HTNV infection, 27.08% (13/48) were with SEOV infection, and 25% (12/48) were untypable. There is a significant difference in the titers of neutralizing antibodies against vaccine strains and epidemic strains detected in the serum samples. For HTNV, both patient and vaccinee sera exhibited significantly higher geometric mean titer (GMT) against vaccine strain 84FLi than against epidemic strain SX26 (1:386.08 vs. 1:223.04, 𝑃 < 0.001; 1:137.94 vs. 1:110.37, 𝑃 < 0.05, respectively). For SEOV, patient sera showed significantly lower GMT against vaccine strain L99 than against epidemic strain LN03 (1:177.02 vs. 1:297.71, 𝑃 < 0.01), and vaccinee sera showed significantly lower GMT against L99 than against epidemic strain FJ-17 (1:97.52 vs. 1:243.71, 𝑃 < 0.001). Significant immunogenicity heterogeneity was observed between SEOV epidemic strains LN03 and FJ-17.

**Conclusions/significance:**

The genetic diversity and immunogenicity of HV, which causes HFRS, have certain geographical regional characteristics. The levels of cross neutralizing antibodies induced by infection of HV from different genotypes may vary significantly, but still provide sufficient cross protection. The HFRSbiv currently used in China could induce broad neutralizing antibody, the cross-protective efficacy might vary against different circulating strains and genotypes. It is of great significance to strengthen the monitoring of genetic characteristics of regional specific circulating viral strains, regularly evaluate the matching degree between vaccine protection effectiveness and epidemic strains, predict and evaluate the transmission and pathogenic risks of HV, and timely optimize HFRS prevention and control strategies and measures.

## Author summary

Hemorrhagic fever with renal syndrome (HFRS) caused by HV remains a serious threat to global public health security, with its epidemic scope continuing to expand. After more than 20 years of application in China, HFRS bivalent inactivated vaccine (HFRSbiv) has been proven to effectively prevent HFRS. However, the evolution and mutation of the virus pose challenges to this specific prevention and control measure. In this study, the levels of neutralizing antibodies against the vaccine strains and the current circulating virus strains in the panel of sera collected from confirmed HFRS patients at convalescent phase and adults who had received standardized immunization with HFRSbiv were evaluated. There are significant differences in the immunogenicity of cross protection induced by locally prevalent HV strains and vaccine strains (HTNV 84FLi and SEOV L99), which suggested that the effectiveness of HFRSbiv may vary in different parts of China. Our findings highlight the importance of the monitoring of genetic characteristics and immunogenicity of regional specific circulating viral strains to predict and evaluate the transmission and pathogenic risks for timely optimization of HFRS prevention and control strategies and measures.

## Introduction

Several rodent-borne hantaviruses are recognized as important human pathogens causing hemorrhagic fever with renal syndrome (HFRS) primarily in Asia and Europe, and hantavirus pulmonary syndrome (HPS) in the Americas, which are mainly acquired through the inhalation of infectious respiratory particles from rodent urine or feces. The viruses are distributed globally, and approximately 200,000 cases are reported annually, posing a persistent threat to human health [1]. In China, Hantaan virus (HTNV) and Seoul virus (SEOV) are the causative agents of HFRS, and the infection has a relatively wide distribution, with the highest annual reported cases exceeding 110000 in 1980s [2]. Although the annual incidence of HFRS had significantly decreased and maintained a low level in the last two decades, the geographical distribution range of Hantavirus (HV) infection had shown an expanding trend, and the virus presents a more abundant genetic diversity [3].

HV are predominantly hosted by rodents and exhibit strict host specificity, each hantavirus species is primarily carried and transmitted by one or several closely related reservoir hosts [4]. Virus–host adaptive evolution is influenced by ecological environments, host switching events, and gene selection pressures, contributing to the genetic diversity of hantaviruses. Moreover, as segmented RNA viruses, HV possess the capacity for genetic reassortment, enabling the exchange of genomic segments between two parental viruses co-infecting the same cell, thereby generating progeny viruses genetically distinct from both parents [5]. The frequency of segment exchange and the co-infection rate within the same host cell affect the success of genomic reassortment [6]. With the rapid development of laboratory testing technology and the expansion of pathogen monitoring scope, new HV strains continued to be discovered, presenting rich genetic diversity with regional characteristics in China [3,7].

It has been demonstrated that immunization with HFRS bivalent inactivated vaccine (HFRSbiv) which contains HTNV and SEOV, was an effective measure for HFRS prevention. HFRSbiv targeting HTNV and SEOV had been licensed for over two decades. Combined with comprehensive interventions such as immunization, rodent control and environmental sanitation, the disease burden of HFRS in China has been significantly reduced [8,9]. However, the vaccine strains used in China (e.g., HTNV PS-6, SD9805, and SEOV L99, HB9908) were isolated more than 20 years ago. Antigenic variation may exist among different strains within the same serotype, and its impact on vaccine protective efficacy remains unclear. Therefore, evaluating the immunogenicity of regional epidemic HV strains, as well as the immunogenic matching and cross-protection between vaccine strains and currently circulating strains, is practically important.

In this study, a serum panel was established using serum samples collected from confirmed HFRS patients and HFRS vaccinees for a comparative analysis of the immunogenicity of HV circulating in China and vaccine strains using an enzyme-linked immunosorbent assay-based microneutralization test (MN-ELISA). The neutralizing antibody levels against vaccine strains and representative epidemic strains were systematically compared to elucidate immunogenicity differences and to provide experimental evidence for the scientific evaluation of the protective efficacy of HFRS vaccine.

## Materials and methods

### Ethics statement

This study received ethics approval from the Ethical Committee of National Institute for Viral Disease Control and Prevention, Chinese Center for Disease Control and Prevention. Written informed consent was obtained from all participants.

### Study subjects

A total of 48 convalescent serum samples from HFRS patients and 28 serum samples from individuals who had completed a three-dose regimen with HFRSbiv were collected between 2019 and 2020 from Heilongjiang Province, which belonged to HFRS national surveillance system. The median interval between disease onset and serum collection for HFRS patients was 14 days, with an interquartile range (IQR) of 10.5 to 19.5 days. Serum samples from vaccinees were collected within one month after the third (booster) dose.

### HV amplification and titration

The HTNV vaccine strain 84FLi, SEOV vaccine strain L99, and previously isolated epidemic strains SX26, LN03, and FJ-17 from HFRS national surveillance system were selected for neutralization assays.

All strains were propagated in Vero-E6 cells prior to use. Viral replication was confirmed by quantitative real-time reverse transcription polymerase chain reaction (qRT-PCR) and indirect immunofluorescence assay (IFA). Viral titers were determined by plaque assay.

#### Virus resuscitation, propagation, and identification.

HV vaccine strains (84FLi, L99) and epidemic strains (SX26, LN03, FJ-17) were thawed on ice and used to infect Vero-E6 cells. Culture supernatants were collected on day 7 post-infection for HV nucleic acid detection by qRT-PCR. Concurrently, cell antigen slides were prepared for IFA.

#### Plaque assay for viral titration.

Vero-E6 cells cultured for 24 h were seeded into 6-well plates and incubated overnight at 37°C with 5% CO_2_. After forming confluent monolayer, the monolayers were washed, and 500 μL of MEM maintenance medium was added to each well. Serial 10-fold dilutions (10^−1^ to 10^−5^) of virus stocks were prepared in MEM, and 500 μL of each dilution was inoculated per well in duplicate. Plates were incubated at 37°C, 5% CO_2_ for 1.5–2 h for adsorption. After removal of the inoculum, 3 mL of pre-warmed (42°C) agarose overlay (mainly 2 x MEM containing 10% fetal bovine serum,2% Gibco HEPES,1% Penicillin-Streptomycin and 2% low-melting-point agarose) was added per well. Once solidified, plates were inverted and incubated at 37°C, 5% CO_2_. Seven days later, a second agarose overlay, which was identical to that of the first overlay, with the addition of 3% neutral red to facilitate plaque visualization, was added, and incubation continued for an additional 48–72 h. Plaques were observed and counted. Viral titers were calculated as plaque-forming units per milliliter (PFU/mL): PFU/mL = (average plaque count at a given dilution × reciprocal of dilution) / virus inoculum volume (mL/well).

### Detection of serum HV IgG antibodies

HV IgG antibodies in all serum samples were detected using a commercial HV IgG antibody ELISA kit (Beijing Beier Bioengineering Co., Ltd., enzyme immunoassay). Positive and negative controls were included. All procedures and result interpretation were performed strictly in accordance with the manufacturer’s instructions.

### Neutralizing antibody assay (MN-ELISA)

A fixed-virus (100 PFU/0.1 mL) microneutralization assay was employed. Sera were heat-inactivated at 56°C and serially diluted 2-fold in 96-well plates (starting dilution 1:10 for vaccinees and 1:20 for patients, ending at 1:10240), with three replicates per dilution. Each virus strain (84FLi, L99, SX26, LN03, FJ-17) was diluted to working concentration, and 50 μL of virus was mixed with an equal volume of diluted serum. The mixtures were incubated at 37°C, 5% CO_2_ for 1 h. Subsequently, 100 μL of Vero-E6 cell suspension was added to each well. Virus-positive and cell-negative controls were included. After seven days of incubation at 37°C, 5% CO_2_, supernatants were discarded, and cells were fixed with cold acetone and inactivated under UV light. After washing, 100 μL of detection antibody mixture (HV monoclonal antibody L13F3 at 1:3200 dilution and HRP-conjugated rabbit anti-mouse IgG at 1:1000 dilution, pre-mixed) was added per well and incubated at 37°C for 45 min. After washing, 100 μL of the mixed TMB substrate solution was added to each well and incubated for 10 min at room temperature in the dark. The reaction was stopped by adding 50 𝜇L of 2 M H_2_SO_4_ per well, and the optical density (OD) was measured at 450 nm using a microplate reader.

### Determination of neutralizing antibody titer and virus typing

The neutralizing antibody titer was defined as the highest serum dilution that inhibited 50% of viral infection. The cutoff value (X) for positivity was calculated as: X = [(mean OD of cell-positive controls – mean OD of cell-negative controls)/2) + mean OD of cell-negative controls [10]. Samples with OD values below X were considered positive for neutralization, and the reciprocal of the highest dilution showing positivity was taken as the neutralizing antibody titer.

For virus typing, the neutralizing antibody titers against the HTNV vaccine strain 84FLi and the SEOV vaccine strain L99 were compared. If the titer against one type was ≥ 4-fold higher than that against the other, the infection was assigned to that type; otherwise, it was considered untypable.

### Statistical analysis

Raw data were entered, organized, and analyzed using Excel 2019 and GraphPad Prism 10.1.2. Neutralizing antibody titers were expressed as geometric mean titer (GMT). Comparisons among multiple groups were performed using one-way analysis of variance (ANOVA). For non-normally distributed data, non-parametric tests (Wilcoxon signed-rank test for two-group comparisons, Friedman test for paired multiple-group comparisons) were applied. A 𝑃-value < 0.05 was considered statistically significant.

## Results

### HV amplification and titer determination

To obtain working virus stocks for subsequent neutralization assays, five HV strains (vaccine strains: 84FLi, L99; epidemic strains: SX26, LN03, FJ-17) were resuscitated, propagated, and titrated. All strains replicated efficiently in Vero-E6 cells. On day 7 post-infection, culture supernatants were collected and tested by qRT-PCR, all yielding positive results. IFA revealed typical bright green specific fluorescent granules in infected cells, which were absent in negative control cells, confirming intact viral infectivity (Fig 1). Viral titers were determined by plaque assay. All strains formed classical plaques at a 10^−3^ dilution (Fig 2). The calculated viral titers (PFU/mL) were as follows: 84FLi, 3.08 × 10^6^; L99, 2.25 × 10^6^; SX26, 5.13 × 10^5^; LN03, 1.23 × 10^4^; FJ-17, 1.65 × 10^6^. These results confirmed successful amplification of all strains for subsequent neutralization assays.

**Fig 1:**
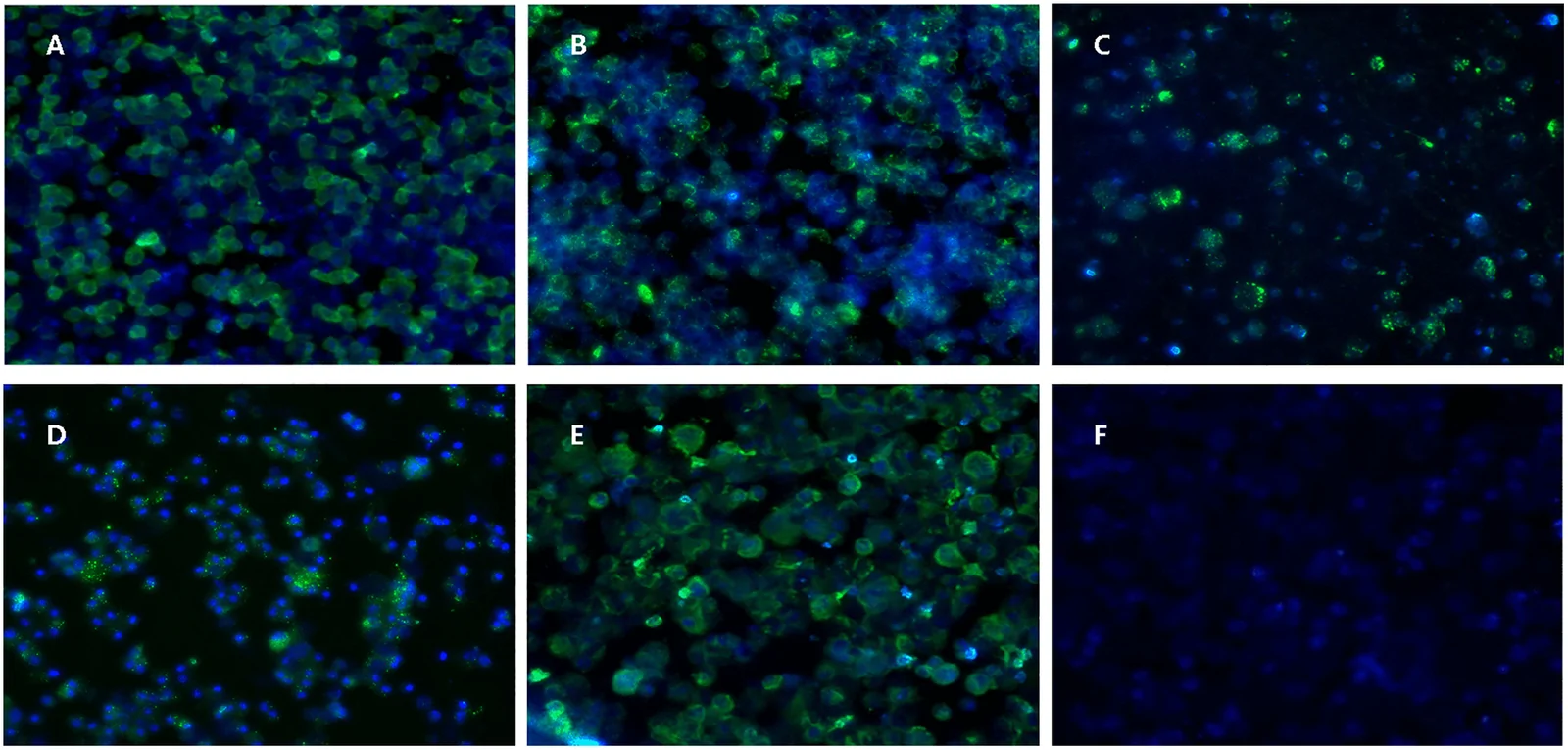
Detection of HV infection in Vero-E6 cells by indirect immunofluorescence assay (IFA). Note: The observations were performed at ×200 magnification. (A) 84FLi; (B) L99; (C) SX26; (D) LN03; (E) FJ-17; (F) Negative control cells.

**Fig 2:**
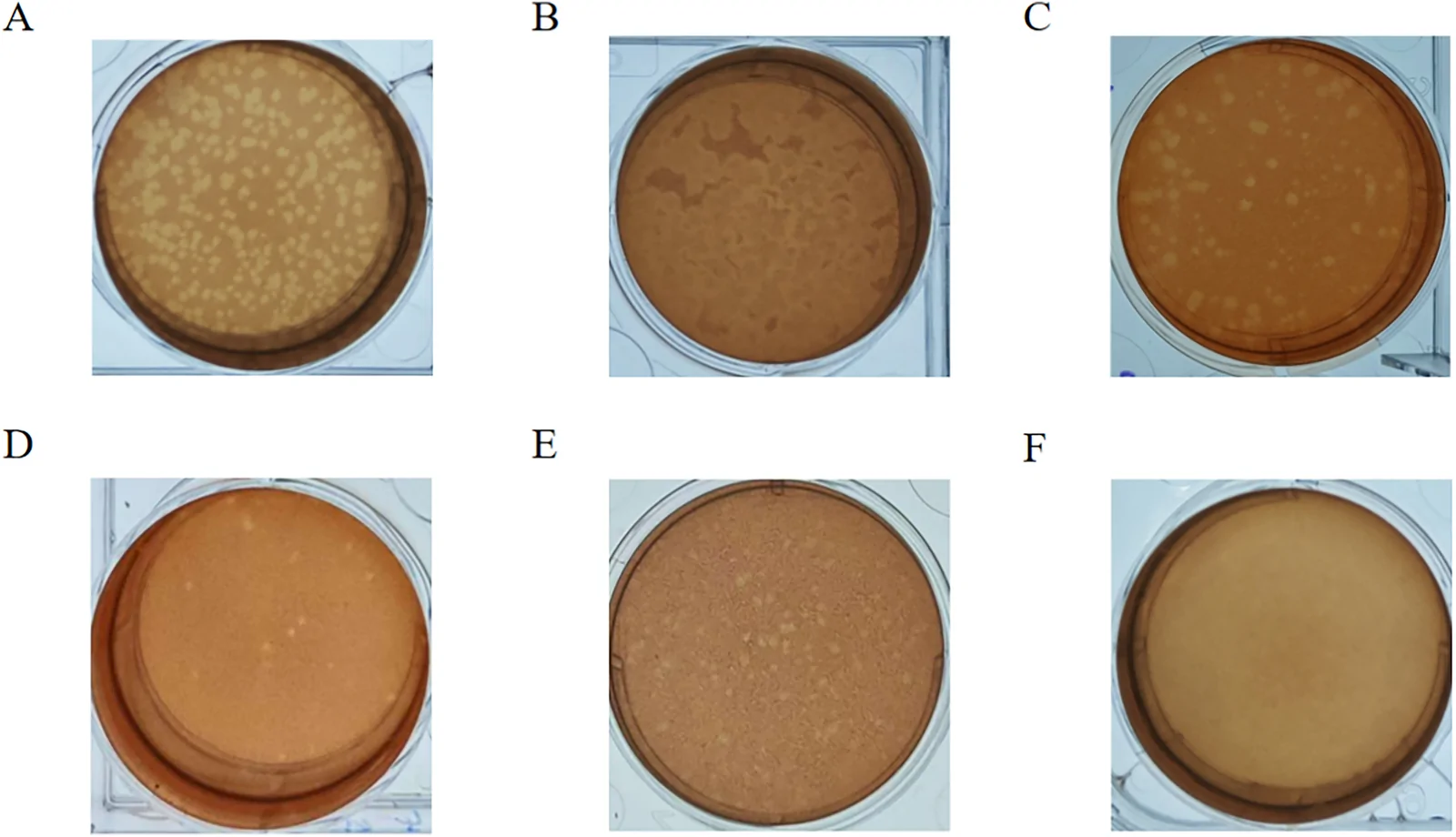
Plaque morphology of HV strains at 10^-3^ dilution. Note: plaque assays were performed in 6-well plates (well diameter: 35 mm). (A) 84FLi; (B) L99; (C) SX26; (D) LN03; (E) FJ-17; (F) Negative control.

### Demographic characteristics and HV typing of HFRS patients

Among the 48 HFRS patients, 38 were male and 10 were female, with a median age of 53 years. Farmers accounted for 83.33% (40/48) of the cases; the remaining occupations included three students, one worker, one retiree, and three unemployed/homemakers. Among the 28 vaccinees (11 farmers, 11 students, and 6 teachers), 9 were male and 19 were female, with a median age of 31 years. Sera from HFRS patient and vaccinees were all tested positive for HV IgG antibodies.

Based on the typing criteria, of the 48 HFRS patient sera, 23 (47.92%) were identified as HTNV infection, 13 (27.08%) were SEOV infection, and the remaining 12 sera were untypable.

### Inter-individual variation in neutralizing capacity against HV vaccine and epidemic strains

Among the 48 HFRS patient sera, 33 showed higher neutralizing titers against HTNV vaccine strain 84FLi than against epidemic strain SX26, with most differences within 2-fold. Ten sera (20.83%, 10/48) exhibited ≥4-fold higher titers against 84FLi than against SX26. In contrast, neutralizing titers against the SEOV vaccine strain L99 were generally lower than those against the epidemic strain LN03. Nearly half of the sera showed ≥4-fold higher titers against LN03 than against L99. Furthermore, immunogenicity differed between the two SEOV epidemic strains LN03 and FJ-17; 83.33% (40/48) of sera exhibited higher neutralizing titers against LN03 than against FJ-17. Most sera showed low neutralizing titers against FJ-17, with nearly half (43.75%) having titers <1:20 (Fig 3). These findings suggest that antigenic differences among HV epidemic strains from different regions may influence cross-protective efficacy.

**Fig 3:**
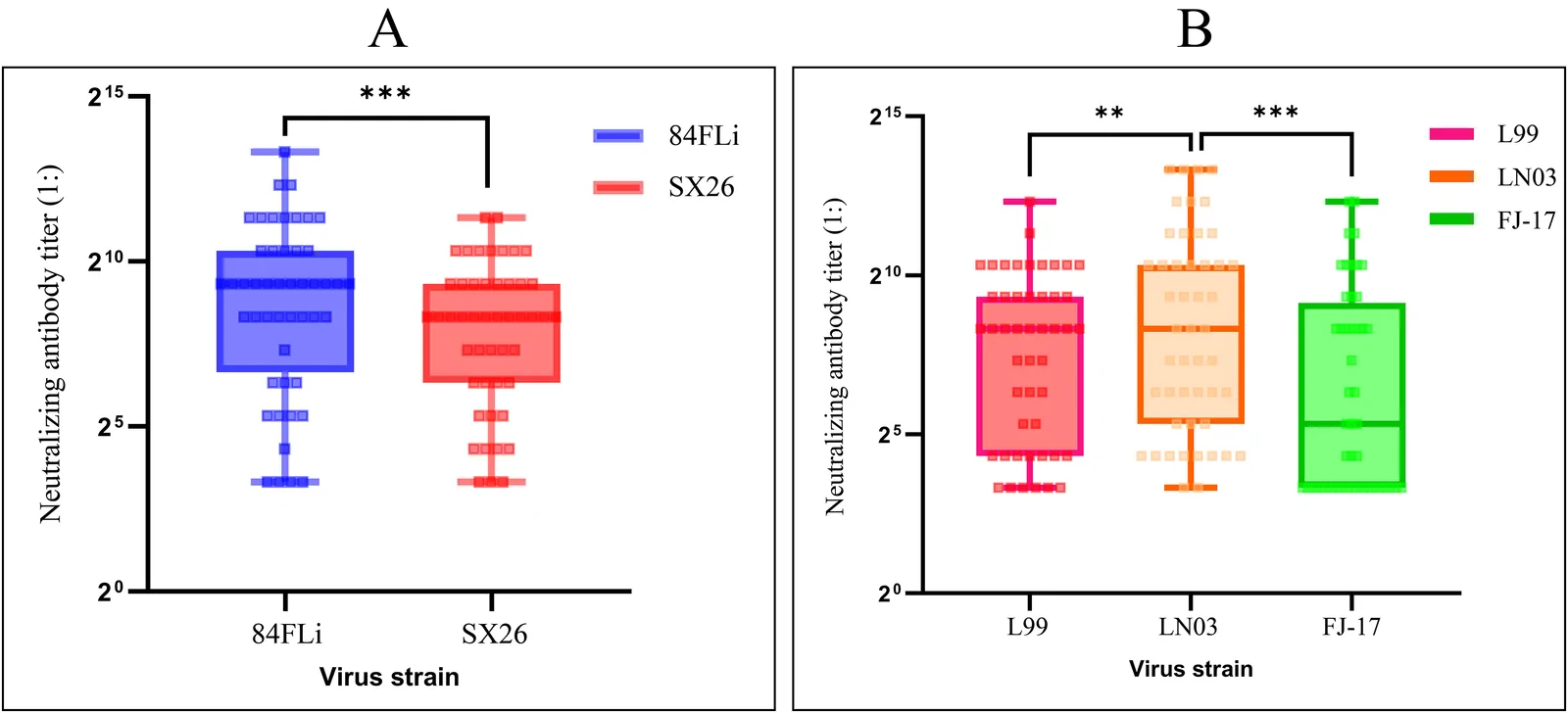
Neutralizing antibody titers of convalescent patient sera against HV strains. (A) HTNV strains; (B) SEOV strains. Note: ** *P* < 0.01, *** *P* < 0.001.

According to a previous study by China Food and Drug Administration [11], a neutralizing antibody titer n [1a Food and Drug Administration [1st HV straAll 28 vaccinee sera tested positive for neutralizing antibodies against 84FLi, SX26, L99, LN03, and FJ-17, with titers ranging from 1:10–1:2560 (Fig 4), indicating robust immunogenicity of HFRSbiv in China. Neutralizing antibody titers against HV vaccine strains and representative epidemic strains by MN-ELISA are presented in S1 Table.

**Fig 4:**
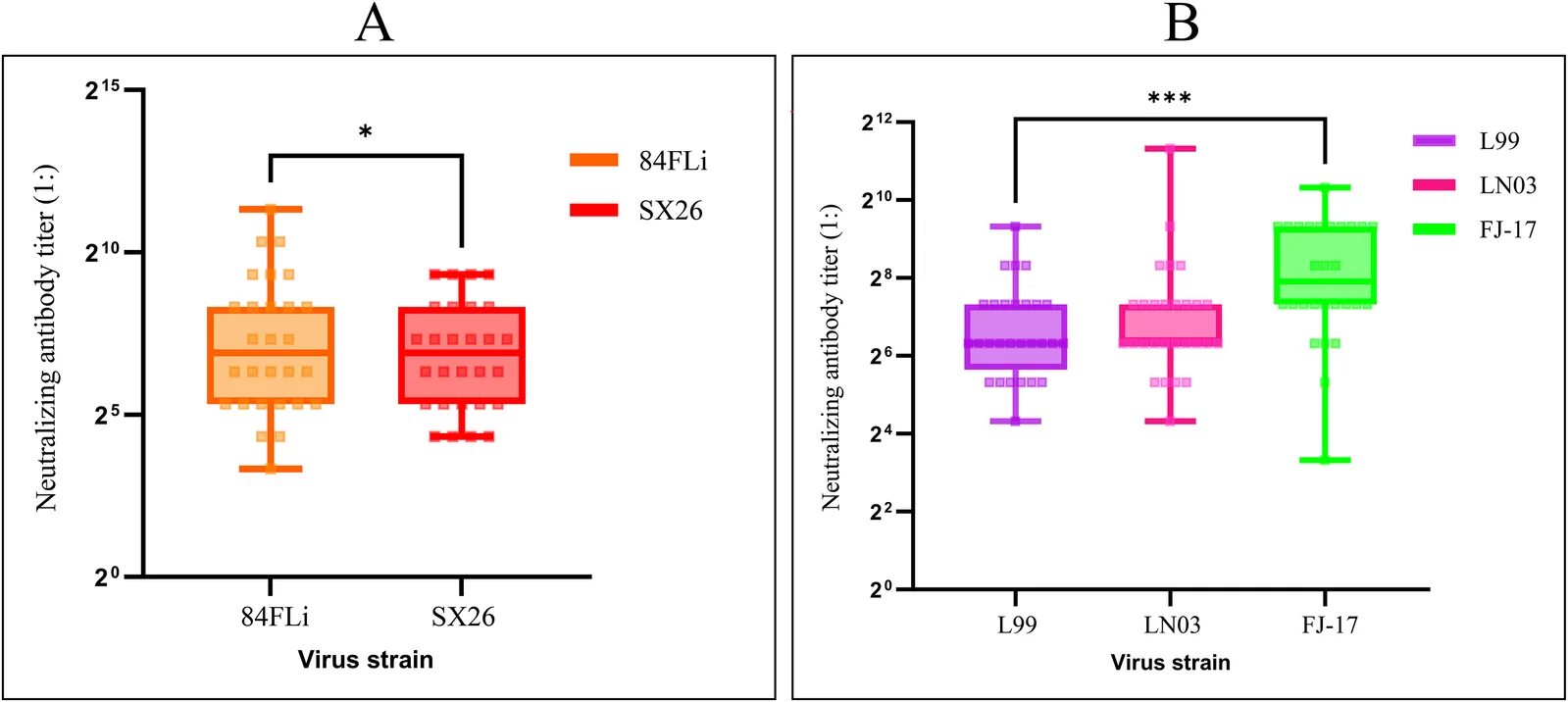
Neutralizing antibody titers of vaccinee sera against HV strains. (A) HTNV strains; (B) SEOV strains. Note: * *P* < 0.05, *** *P* < 0.001.

### Population-level neutralizing antibody responses against HV vaccine and epidemic strains

The GMT of neutralizing antibodies against the HTNV vaccine strain 84FLi in 48 HFRS patient sera was 1:386.08, which was 1.73-fold higher than that against the epidemic strain SX26 (GMT = 1:223.04) (𝑃 < 0.001). The GMT against the SEOV vaccine strain L99 was 1:177.02, which was 40.54% lower than that against the epidemic strain LN03 (GMT = 1:297.71) (𝑃 < 0.01), and 2.34-fold higher than that against FJ-17 (GMT = 1:75.52) (𝑃  > 0.05). Moreover, the GMT against LN03 was 3.94-fold higher than that against FJ-17 (𝑃 < 0.001).

Among the 28 vaccinee sera, the GMT against the HTNV vaccine strain 84FLi was 1:137.94, compared to 1:110.37 against the epidemic strain SX26 (1.25-fold difference). The GMT against the SEOV vaccine strain L99 was 1:97.52, which was 59.99% lower than that against the epidemic strain FJ-17 (GMT = 1:243.71) (𝑃 < 0.001), 17.97% lower than that against LN03 (1: 118.89) (𝑃 > 0.05) (Table 1).

**Table 1:** Neutralizing antibody titers against HV vaccine strains and epidemic strains in different study populations.

Serum Source	Sample Size (n)	GMT (95% CI*) of Neutralizing Antibodies (1:)				
		84FLi	SX26	L99	LN03	FJ-17
HFRS Patients	48	386.08 (229.57 – 649.23)	223.04 (143.16 – 347.42)	177.02 (104.02 – 301.28)	297.71 (159.87 – 554.40)	75.52 (40.30 – 141.44)
Vaccinees	28	137.94 (79.65 – 238.85)	110.37 (70.94 – 171.64)	97.52 (71.58 – 132.83)	118.89 (81.62 – 173.16)	243.71 (160.76 – 369.50)

Note: *CI = confidence intervals.

## Discussion

It is well known that vaccination is an effective public health intervention for reducing the morbidity of infectious diseases. Since the 1990s, vaccination had become a key measure for HFRS prevention and control in China, not only reducing the risk of individual infection but also contributing to the suppression of viral transmission and epidemic spread to a certain extent [12]. The HFRSbiv in China is administered to populations in key regions and high-risk groups. It induces both specific IgG antibodies and neutralizing antibodies, and the short-to-medium-term protection was approximately 95% [13–15]. Neutralizing antibody levels are considered direct serological correlates of vaccine-induced protective efficacy [16]. However, the vaccine strains (e.g., HTNV 84FLi, PS-6, and SEOV L99) were isolated more than two decades ago. HV continue to evolve in nature, exhibiting marked genetic diversity and geographic clustering [7,17]. Whether antigenic drift [18] has occurred in the viral subtypes circulating in different provinces, and whether such drift affects the cross-protective efficacy of current vaccines, has become a critical scientific question requiring urgent evaluation for HFRS control in China.

Here, we used five strains, including 84FLi (HTNV genotype 5), L99 (SEOV genotype 1), and LN03 (SEOV genotype 3), which are the most widely distributed HTNV and SEOV genotypes in China [3], to evaluate immunogenicity of the HFRS vaccine. Of that, SX26 was isolated from Shaanxi Province in 2015, which belongs to HTNV serotype (genotype 4) and currently is predominant in central and northwestern China. In the glycoprotein encoded by the M segment, sequence alignment identified three amino acid variant sites compared with vaccine strain 84FLi. LN03 belonged to SEOV (genotype 3) was isolated from Liaoning Province in 2020, northeastern China, where genotype 3 SEOV have been endemic [17]. SEOV strain FJ-17 was isolated from Fujian Province, southeastern coastal China in 2017, and belongs to the divergent SEOV genotype 5 [17]. The M segment of FJ-17 shares only 84.3% nucleotide acid identity with the vaccine strain L99. The selection of HV strains were based on their availability, geographical representation and genetic divergence from the vaccine strains [3], which may lead to potential antigenic changes.

In this study, we systematically compared neutralizing antibody responses against vaccine strains and currently circulating epidemic strains using sera from HFRS convalescent patients and HFRSbiv recipients via MN-ELISA. For HTNV, both patient and vaccinee sera exhibited higher GMT against the vaccine strain 84FLi than against the same serotype epidemic strain SX26 (1.73-fold and 1.25-fold, respectively). This suggests that the HTNV vaccine strain still induces effective neutralizing antibodies against the circulating strains, but also implies potential antigenic changes in SX26 that result in slightly reduced neutralization sensitivity. Such differences may arise from adaptive evolution of epidemic strains under long-term vaccination pressure or from antigenic advantages conferred by vaccine strains during cell culture passage.

In contrast, the SEOV results revealed a more complex pattern. Patient sera exhibited significantly higher GMT against the epidemic strain LN03 than against the vaccine strain L99 (1.68-fold ratio), while neutralizing capacity against another epidemic strain, FJ-17, was generally weaker. Among vaccinees, GMT against FJ-17 was even significantly higher than those against L99 (2.50-fold ratio). These data indicate substantial heterogeneity in immunogenicity among different SEOV strains. LN03 (SEOV genotype 3) is the predominant strain in the northeastern provinces of China (Heilongjiang, Jilin, Liaoning) [17,19] and shows good antigenic matching with antibodies induced by natural infection in that region. Conversely, FJ-17 belongs to SEOV genotype 5, which is primarily distributed along the southeastern coast of China and exhibits considerable phylogenetic divergence from other domestic and international SEOV reference strains [20]. Previous studies [21,22] showed a S882I substitution in the Gc protein of FJ-17 within a neutralizing epitope region. This substitution may alter epitope exposure or protein conformation, thereby enabling cross-reactive antibodies to achieve stronger functional recognition of FJ-17. The differences in GMT of neutralizing antibodies induced by L99, LN03, and FJ-17 suggest that sera from immunized individuals remain capable of neutralizing the epidemic HV strain of a different genetic subtype, and that the immune response elicited by the SEOV‑type vaccine strain has not undergone substantial changes in its activity against epidemic strain infection.

Furthermore, previous study showed that both HTNV and SEOV possess mutation sites, with SEOV exhibiting substantially fewer variant sites than HTNV [17]. Notably, several variations in glycoprotein epitopes (V442I, V996I) have been identified in the SX26 strain compared to the HTNV vaccine strain 84FLi, which suggested that potential variations in glycoprotein epitopes, possibly due to sequence differences among the strains, may contribute to the observed antigenic differences of HV. However, detailed amino acid mapping is required to be studied further.

Taken together, our study demonstrates that HFRSbiv currently used in China induces broad neutralizing antibody responses. However, its cross-protective capacity varies against different epidemic strains and genotypes across regions. The HTNV vaccine component maintains relatively good immune coverage against currently circulating strains, whereas the SEOV vaccine component faces greater challenges due to antigenic diversity, with potentially region-specific protective efficacy. These findings underscore the importance of continuous monitoring of vaccine strain–epidemic strain matching and the need to periodically evaluate and optimize vaccination strategies.

This study has several limitations. The 25% (12/48) untypable rate likely reflects cross-neutralization between HTNV and SEOV. In addition, this typing result depends on the representative strains used and may not fully capture the overall picture of cross-reactivity. Given the genotype diversity of HV in China and the narrow geographical origin of the samples, the thorough analysis of immunogenicity against HTNV and SEOV could be compromised. The HFRS patients and vaccinee were not comparable in age and sex distribution, which may confound direct comparisons of immune responses. The serum panel was relatively small, particularly for the vaccinee cohort. Future studies should expand geographic coverage and sample size, integrate viral genomics and structural biology approaches to elucidate the molecular basis of antigenic variation, and explore the necessity and feasibility of vaccine strain updates or multivalent vaccine development. Such efforts will provide a scientific foundation for more effective long-term HFRS prevention and control strategies.

## Supporting information

S1 Table Neutralizing antibody titers against HV vaccine strains and epidemic strains by MN-ELISA. (PDF)
