# Supplementary material for: Immunogenicity of Hantavirus and evaluation of vaccine protective efficacy: A comparative neutralizing antibody study of vaccine strains and representative epidemic strains from selected provinces in China
Source: PLoS Negl Trop Dis. 2026 Nov 11;20(11):e0014811. doi: 10.1371/journal.pntd.0014811 (PMC13754224; doi:10.1371/journal.pntd.0014811)
Supplement: Supplementary file 1 [file pntd.0014811.s001.pdf]

S1 Table Neutralizing antibody titers against HV vaccine strains and epidemic strains by MN-ELISA

No.	HTNV (1:X)		SEOV (1:X)		
	84FLi	SX26	L99	LN03	FJ-17
1	80	80	＜ 20	＜ 20	＜ 20
2	640	2560	640	1280	640
3	80	40	20	＜ 20	＜ 20
4	5120	1280	320	2560	320
5	20	＜ 20	160	640	1280
6	640	640	＜ 20	160	40
7	1280	1280	640	10240	1280
8	320	320	40	640	80
9	160	320	80	1280	640
10	640	320	20	40	＜ 20
11	2560	1280	20	1280	＜ 20
12	640	320	＜ 20	2560	20
13	＜ 20	＜ 20	320	10240	320
14	640	320	640	640	1280
15	1280	640	＜ 20	80	＜ 20
16	1280	640	20	160	40
17	320	80	＜ 20	160	＜ 20
18	40	20	320	1280	640
19	10240	2560	640	5120	1280
20	320	80	20	20	＜ 20
21	40	40	640	1280	320
22	40	80	2560	10240	2560
23	640	320	20	320	20
24	80	40	320	1280	320
25	320	160	20	20	＜ 20
26	640	320	＜ 20	40	320
27	320	160	5120	10240	5120
28	2560	1280	80	80	40
29	2560	640	320	40	＜ 20
30	640	640	160	160	＜ 20
31	40	20	1280	2560	＜ 20
32	2560	640	1280	20	＜ 20
33	320	160	1280	20	＜ 20
34	640	320	640	80	＜ 20
35	2560	1280	1280	20	＜ 20
36	640	640	1280	80	＜ 20
37	1280	160	1280	2560	＜ 20
38	1280	320	160	20	＜ 20
39	640	320	320	20	＜ 20
40	＜ 20	＜ 20	320	80	80
41	＜ 20	20	320	320	160
42	320	320	1280	20	＜ 20
43	5120	1280	640	320	1280
44	＜ 20	20	320	640	320
45	320	160	40	80	＜ 20
46	2560	640	1280	5120	5120
47	640	320	80	1280	320
48	2560	1280	1280	5120	2560
49	40	40	80	160	320

50	40	40	40	160	640
51	640	640	80	160	640
52	80	40	160	160	160
53	40	20	40	80	640
54	320	160	40	40	10
55	10	320	80	80	40
56	20	20	80	80	640
57	1280	640	640	2560	1280
58	40	40	160	320	160
59	20	20	40	80	640
60	640	640	80	160	160
61	160	160	160	320	160
62	320	160	320	640	640
63	160	160	80	160	160
64	160	80	160	160	320
65	80	320	160	320	160
66	320	320	40	40	160
67	80	20	160	80	320
68	80	80	80	40	160
69	40	40	40	40	80
70	320	160	320	80	640
71	2560	160	80	80	640
72	640	320	320	160	640
73	40	80	20	20	80
74	1280	640	160	80	640
75	320	80	80	80	80
76	80	80	80	80	160
